# Uniportal video-assisted thoracic surgery lowers the incidence of post-thoracotomy pain syndrome

**DOI:** 10.20407/fmj.2019-008

**Published:** 2020-02-11

**Authors:** Hiromitsu Nagano, Takashi Suda, Hisato Ishizawa, Takahiro Negi, Hiroshi Kawai, Toru Kawakami, Daisuke Tochii, Sachiko Tochii, Yasushi Hoshikawa

**Affiliations:** Department of Thoracic Surgery, Fujita Health University, School of Medicine, Toyoake, Aichi, Japan

**Keywords:** Uniportal VATS, Post-thoracotomy pain syndrome, Minimally invasive surgery, Surgery

## Abstract

**Objective:**

We compared post-thoracotomy pain syndrome (PTPS) incidence in patients who underwent uniportal or multiportal video-assisted thoracoscopic surgery (VATS).

**Methods:**

We included 223 patients who underwent either uniportal or multiportal VATS between January 2017 and October 2018 (pulmonary lobectomies and pulmonary segmentectomies—uniportal: *n*=19, multiportal: *n*=133; wedge lung resections—uniportal: *n*=16, multiportal: *n*=55). We retrospectively studied incidences of PTPS in all subgroups.

**Results:**

Incidences of PTPS were significantly less for uniportal procedures for both the pulmonary lobectomy/segmentectomy group (*P*=0.024) and the wedge lung resection group (*P*=0.0315) than for multiportal procedures.

**Conclusion:**

Patients who underwent uniportal VATS procedures had lower incidences of PTPS than the multiportal VATS group. The uniportal VATS approach is therefore beneficial for patients.

## Introduction

Uniportal thoracoscopic lung resection was first reported by Rocco et al. in 2004 for a wedge resection of the lung.^1^ In 2011, Gonzalez-Rivas et al. reported a pulmonary lobectomy using uniportal video-assisted thoracoscopic surgery (VATS),^2^ after which the use of uniportal VATS expanded, primarily in Asia and Europe. Compared with conventional multiportal thoracoscopic pneumonectomy, uniportal VATS reportedly shortens surgery time, reduces intraoperative bleeding, shortens the thoracostomy tube indwelling period and hospitalization period, reduces complication incidence, alleviates postoperative pain, and improves esthetic outcomes.^3–5^

Post-thoracotomy pain syndrome (PTPS) has been previously reported,^6^ and defined by the International Association for the Study of Pain as refractory pain along a surgical wound, which persists for least 2 months after surgery or relapses at least 2 months after surgery.^7^ The reported incidence of PTPS is 11–80%.^8^ However, few studies have evaluated chronic pain following uniportal VATS.^9^ In this study, we compared PTPS incidence among patients who underwent uniportal or multiportal VATS.

## Materials and Methods

### Subjects

This retrospective study was approved by the Ethics Committee of Fujita Health University. At Fujita Health University Hospital, 516 patients underwent thoracoscopic surgery between January 2017 and October 2018. Among the 382 patients who remained after excluding those who underwent either robot-assisted surgery or thoracotomy, 225 patients underwent pulmonary lobectomy and pulmonary segmentectomy (PLPS) and 157 patients underwent wedge lung resection. To compare chronic pain caused by uniportal and multiportal approaches, we excluded (a) patients who received bilateral thoracoscopic surgery in 1 or 2 stages; (b) patients whose medical follow-up ended less than 2 months after surgery; (c) patients aged <10 years; (d) patients with suspected bone metastasis; and (e) patients who could not be evaluated with numerical rating scale (NRS) pain scores. However, among patients who underwent bilateral thoracoscopic surgery in 1 or 2 stages, the first surgery was included in this study if the interval between the first and second surgeries of a two-stage surgery was more than 2 months. After excluding the abovementioned patients, we included 152 patients who underwent PLPS and 71 patients who underwent wedge lung resections.

**Figure 1:**
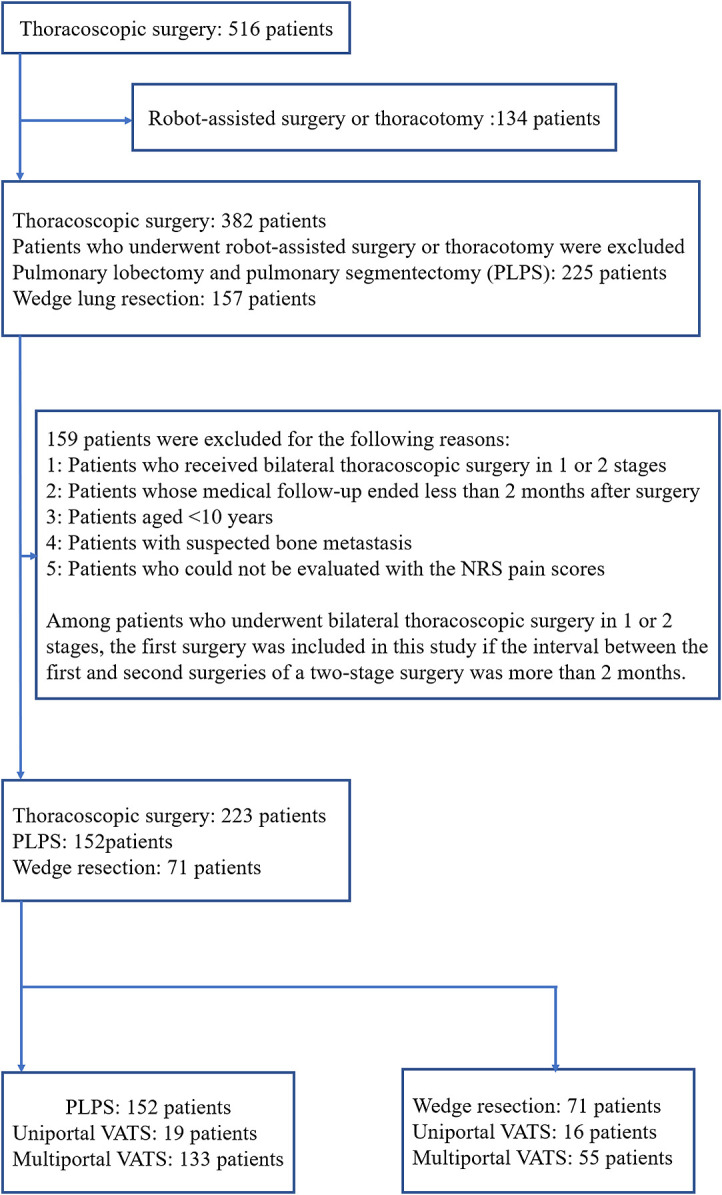
Exclusion criteria

Uniportal VATS was performed only when the operator or 1^st^ assistant was a surgeon skilled in uniportal surgery. Multiportal VATS was performed when the operator or 1^st^ assistant was not a surgeon skilled in uniportal surgery, when an undiagnosed tumor needed to be located by palpation, or when CT-guided needle marking could not be performed. Among the 152 patients who underwent PLPS, 19 had uniportal VATS and 133 had multiportal VATS. Of the 71 patients who underwent wedge resections, 16 had uniportal VATS and 55 had multiportal VATS (Figure 1).

### Surgical procedures

Surgeries were performed with patients under general anesthesia. A double-lumen tube was used as the endotracheal intubation tube. Patients were placed in the lateral decubitus position. The operator stood on the patient’s right side, with the assistants and camera operator on the patient’s left side.

Uniportal VATS for PLPS was performed with only a 3–4-cm incision at the mid-axillary line in the 6^th^ intercostal space, regardless of the resected lobe site, for both left and right surgeries. The thoracoscope used was a 5-mm rigid device (Olympus, Tokyo, Japan). LigaSure^TM^ Maryland (Covidien, Mansfield, MA, USA) or HARMONIC^®^ HD1000i (Ethicon Endo-Surgery, NJ, USA) was used in lymphadenectomy; Powered ECHELON FLEX^®^ GST System (Ethicon Endo-Surgery, NJ, USA) or Endo GIA^TM^ Tri-Staple^TM^ (Covidien, Mansfield, MA, USA) was used for pulmonary and bronchial cutting; and Powered ECHELON FLEX^®^ 7 (Ethicon Endo-Surgery, NJ, USA) was used for pulmonary artery and pulmonary vein dissection. A drainage tube was inserted through the surgical incision.

Multiportal VATS was performed with a 3–4-cm incision at the mid-axillary line in the 4^th^ intercostal space, a 1.5-cm incision at the anterior axillary line in the 6^th^ intercostal space, and a 1.5-cm at the posterior axillary line in the 7^th^ intercostal space. An Alexis^®^ Wound Retractor XS (Applied Medical, CA, USA) was inserted into the 3–4-cm incision, while ports were inserted into the other incisions. The thoracoscopic port site was the anterior axillary line in the 6^th^ intercostal space on the right side, and the posterior axillary line in the 7^th^ intercostal space on the left side. The thoracoscope used was a 10-mm rigid device (Olympus, Tokyo, Japan). The drainage tube was inserted through the incision at the 6^th^ intercostal space.

Uniportal VATS for wedge lung resection was performed only with a 2-cm incision at the mid-axillary line in the 6^th^ intercostal space. The thoracoscope and equipment used for lung resection were identical to those used in uniportal VATS for PLPS. The drainage tube was inserted through the surgical incision.

Multiportal wedge-resection VATS was performed with a 0.5–1.5-cm incision at the mid-axillary line in the 4^th^ intercostal space, a 0.5-cm incision at the anterior axillary line in the 6^th^ intercostal space, and a 1.5-cm incision at the posterior axillary line in the 7^th^ intercostal space on the right side. On the left side, the surgery was performed with a 1.5-cm incision at the anterior axillary line in the 6^th^ intercostal space and a 0.5-cm incision at the posterior axillary line in the 7^th^ intercostal space. The thoracoscope used was a 0.5-cm rigid device (Olympus, Tokyo, Japan), and the equipment used was same as that used in multiportal VATS for PLPS. The drainage tube was inserted through the incision at the 6^th^ intercostal space.

### Postoperative course and postoperative pain management

The drainage tube was removed after postoperative day (POD) 2 in patients who underwent pulmonary lobectomy, and after POD 1 in those who underwent wedge lung resection. In our department, the criteria for removal of the tube are (a) absence of lung collapse or pleural effusion in chest X-ray, (b) less than 200 ml of pleural fluid drainage volume on the previous day, and (c) absence of any air leak. After drainage tube removal, patient progress was observed in accordance with the clinical protocol followed in our department; if no complications were noted, patients were discharged on POD 7 after PLPS or on POD 2 after wedge lung resection.

A catheter for a paravertebral nerve block was intraoperatively inserted into all patients who underwent either surgery. The analgesic used was 200 ml of 0.2% ropivacaine hydrochloride hydrate, which was continuously administered at 5 ml/hour after the surgery; analgesic administration was discontinued on POD 2. If the patient had no swallowing difficulties, loxoprofen 60 mg was initiated at three tablets per day, beginning on POD 1. If pain was not alleviated, acetaminophen, celecoxib, tramadol hydrochloride, and/or pregabalin were added. Oral analgesic dose and administration period were determined by the attending physician. We evaluated postoperative pain in outpatient follow-up visits for 2–3 months after surgery. Pain was evaluated by the attending physician using the NRS pain score.

### Clinical data

Prexpiratoryeoperative, intraoperative, and postoperative patient background and data used in pain evaluation were obtained from the electronic medical records maintained at the Fujita Health University. Preoperative patient background information included the following characteristics: age, sex, height, weight, Brinkman Index, presence or absence of diabetes mellitus, presence or absence of chronic obstructive pulmonary disease, presence or absence of interstitial pneumonitis, preoperative forced expiratory volume in 1 second (FEV_1_), pathologic examination findings, pathologic stage, and number of lymph nodes subjected to lymphadenectomy (Table 1). Postoperative patient background information included the following characteristics: duration of surgery, volume of blood loss, incidence of intraoperative complications, duration of hospitalization, duration of *in situ* pleural cavity drain, duration of hospitalization postoperatively, NRS pain scores on day one after surgery, incidence of postoperative complications, repeated surgery, and length of follow-up (Table 2).

For patients who underwent wedge lung resections, we collected preoperative patient background information on age, sex, height, weight, Brinkman Index, presence or absence of diabetes, presence or absence of chronic obstructive pulmonary disease, presence or absence of interstitial pneumonia, forced expiratory volume in 1 second (FEV_1_), and other diseases (Table 3). Postoperative patient data collected were same as those collected for patients who underwent PLPS (Table 4).

With respect to pain, we evaluated the NRS pain score at 2–3 months after surgery, postoperative oral analgesic administration period, postoperative oral analgesic dose, and incidence of PTPS. Postoperative oral analgesic administration period, and postoperative oral analgesic dose were, respectively, the number of days, and prescription dose from POD 1 to the day of final administration. The quantity of oral analgesics administered postoperatively was measured as the total of loxoprofen, acetaminophen, celecoxib, tramadol hydrochloride, and pregabalin. PTPS is defined as refractory pain associated with a surgical wound, which either persists for at least 2 months after surgery or relapses at least 2 months after surgery.^7^ In the present study, patients were diagnosed with PTPS if the NRS pain score at >2–3 months of surgery was >1, and postoperative oral analgesic administration period was >60 days.

### Statistical analysis

We used JMP^®^ Pro 13 (SAS Institute Inc., Cary, NC, USA) for all statistical analyses. Continuous data were summarized as means±standard deviations, and categorical data as percentages. Continuous data were compared using Student’s *t*-test, non-continuous data were compared using the Mann–Whitney U test, and categorical data were compared using the chi-squared test. *P*<0.05 was considered significant.

## Results

Pain evaluation results are summarized in Tables 5 and 6 for PLPS and wedge lung resection. For PLPS patients, mean postoperative oral analgesic dose was significantly lower in the uniportal VATS group (65.58±41.13 tablets) than in the multiportal VATS group (149.33±170.96 tablets; *P*=0.0041). The incidence of PTPS was significantly lower in the uniportal VATS group (3/19 patients, 15.79%) than in the multiportal VATS group (58/133 patients, 43.61%; *P*=0.024). The uniportal and multiportal groups did not significantly differ in NRS pain scores at 2–3 months after surgery (*P*=0.1973) or in postoperative oral analgesic administration periods (*P*=0.0917).

For the wedge lung resection patients, PTPS incidence was significantly lower in the uniportal VATS group (0/16 patients, 0%) than in the multiportal VATS group (13/55 patients, 26.64%; *P*=0.0315). However, the uniportal and multiportal groups did not significantly differ in NRS pain scores at 2–3 months after surgery (*P*=0.0864), postoperative oral analgesic administration period (*P*=0.2043), and postoperative oral analgesic dose (*P*=0.898).

## Discussion

In the present study, PTPS incidence in the uniportal VATS group was significantly lower than that in the multiportal VATS group. Multiportal video-assisted thoracoscopic surgery (VATS) adversely affects multiple intercostal nerves, whereas uniportal VATS adversely affects only one intercostal nerve site. The observed effects of uniportal VATS are less severe than those of multiportal VATS. As such, compared with the use of multiportal VATS, the use of uniportal VATS is thought to result in a lower observed PTPS incidence. Here, PTPS incidence in the uniportal VATS group for patients who underwent PLPS was similar to that reported in previous studies,^10^ whereas PTPS incidence in the uniportal VATS group for patients who underwent wedge lung resection was lower than that in previous studies.^11^ Liu et al. reported no significant difference in pain at 2 months after surgery between patients who underwent uniportal VATS and those who underwent two-port VATS; however, the pain tended to be lower in the uniportal VATS group.^9^ Based on the results of this study, PTPS incidence in patients who underwent uniportal VATS was expected to be lower than that in patients who underwent multiportal VATS. Postoperative pain is reportedly correlated with occurrence of postoperative complications in patients.^12^ Although incidences of postoperative complications did not significantly differ between the uniportal and multiportal VATS groups in the present study, the incidence tended to be lower in the uniportal group than in the multiportal group.

Pain may be evaluated through various methods, such as the NRS, visual analogue scale (VAS), 5-point verbal rating scale, and McGill questionnaire.^6,13,14^ Because of these diverse assessment methods, diagnostic criteria for PTPS are also diverse, and no fixed criteria have been established. In the present study, patients were diagnosed with PTPS if their NRS pain scores were >1 at 2–3 months after surgery, or if their postoperative oral analgesic administration periods were more than 60 days. We included postoperative oral analgesic administration period >60 days in the evaluation of PTPS because continuing analgesics is thought to indicate continuous pain. According to Louis et al., because VAS data are extremely subjective, its use may reflect considerable bias from both patients and clinicians. Therefore, they advocated that analgesic intake should be used as a more objective method to evaluate postoperative pain.^15^ Accordingly, we included postoperative oral analgesic doses and administration periods in assessing postoperative pain. For patients who underwent PLPS, the total dose of oral analgesics was significantly lower, and the postoperative oral analgesic administration period tended to be shorter, in the uniportal VATS group than in the multiportal VATS group. After wedge lung resection, the total dose tended to be lower and the administration period of oral analgesics tended to be shorter in the uniportal VATS group than in the multiportal VATS group. Based on these results, uniportal VATS may be better than multiportal VATS with respect to postoperative pain.

This study has several limitations. First, it was a retrospective study, with relatively few patients in the VATS group. Moreover, specific, standardized evaluators for pain are not yet established; nor did we have clear criteria regarding the types and doses of postoperative analgesics or for dose reduction and discontinuation.^16^ Therefore, pain evaluation in the present study could have been biased. In the future, to confirm the usefulness of uniportal VATS in preventing PTPS, clear criteria for pain evaluation should be established. Randomized controlled trials, and studies that generate higher quality evidence, should also be conducted.

This study investigated PTPS incidence in patients who underwent uniportal or multiportal VATS for PLPS, or wedge lung resection. The incidence of PTPS in patients who underwent uniportal VATS was lower than that in patients who underwent multiportal VATS. Uniportal VATS procedures are therefore beneficial for patients.

## Figures and Tables

**Table1:** Preoperative background information of patients who underwent uniportal or multiportal lobectomies and segmentectomies with VATS

Characteristic	Uniportal *N*=19	Multiportal *N*=133	*P*
Age (year)	66.00±13.93	68.40±9.14	0.4737
Sex (male/female)	11/8	81/53	1.0000
Height (cm)	160.05±6.24	160.50±8.25	0.7814
Weight (kg)	58.35±8.98	59.69±11.11	0.5601
Brinkman Index	356.58±439.34	625.37±649.10	0.1214
Comorbidity			
Diabetes	4 (21.05)	28 (20.90)	1.0000
COPD	0 (0.00)	7 (5.22)	0.5975
IP	0 (0.00)	1 (0.75)	1.0000
Preoperative FEV1.0 (L)	2.44±0.57	2.31±0.62	0.3607
Pathology			0.4158
Adenocarcinoma	16 (84.21)	96 (72.18)	
Squamous cell carcinoma	1 (5.26)	20 (15.04)	
Small cell carcinoma	0 (0.00)	5 (3.76)	
Large cell carcinoma	0 (0.00)	1 (0.75)	
Pleomorphic carcinoma	0 (0.00)	1 (0.75)	
Carcinoid tumors	0 (0.00)	1 (0.75)	
Epithelioid angiosarcoma	0 (0.00)	1 (0.75)	
Metastatic lung cancer	1 (5.26)	5 (3.76)	
Benign tumors	0 (0.00)	3 (2.25)	
Bronchocystic cyst	1 (5.26)	0 (0.0)	
Pathological stage			0.6617
0	3 (15.79)	8 (6.02)	
IA1	1 (5.26)	15 (11.28)	
IA2	8 (42.11)	38 (28.57)	
IA3	1 (5.26)	20 (15.04)	
IB	1 (5.26)	11 (8.27)	
IIA	0 (0.00)	9 (6.77)	
IIB	2 (10.53)	10 (7.52)	
IIIA	1 (5.26)	12 (9.02)	
IIIB	0 (0.00)	1 (0.75)	
IVA	0 (0.00)	1 (0.75)	
Number of lymph nodes	14.78±7.95	15.37±6.89	0.7790

Data are presented as *n* (%); or as median value±standard error. COPD: Chronic obstructive pulmonary disease, IP: Interstitial pneumonia, FEV1.0: Forced expiratory volume in 1 second; VATS: video-assisted thoracoscopic surgery.

**Table2:** Intraoperative and postoperative data for patients who underwent lobectomy and segmentectomy with VATS

Variable	Uniportal *N*=19	Multiportal *N*=133	*P*
Duration of surgery (min)	170.26±39.39	176.91±52.18	0.6740
Volume of hemorrhage (ml)	45.74±40.01	61.97±90.00	0.7254
Intraoperative complication	0 (0.00)	2 (1.50)	0.8652
Duration of hospitalization (day)	10.11±3.03	12.67±9.08	0.4687
Duration of in situ pleural cavity drain (day)	3.32±2.24	3.59±3.37	0.5971
Postoperative hospital stay (day)	7.84±3.11	10.69±9.44	0.3440
NRS on day one after surgery	3.05±2.50	2.85±2.08	0.8610
Postoperative complication	3 (15.79)	29 (21.80)	0.5686
Atelectasis	1	3	
Pulmonary fistula	1	7	
Arrhythmia	1	8	
Pneumonia	0	8	
Pulmonary embolism	0	1	
Chylothorax	0	1	
Other	0	4	
Repeated surgery	0 (0.00)	2 (1.50)	1.0000
Duration of follow-up (day)	335.78±179.07	497.13±184.75	0.0013

Data are presented as *n* (%); or as median value±standard error. NRS: Numerical Rating Scale; VATS: video-assisted thoracoscopic surgery.

**Table3:** Preoperative background information of patients who underwent uniportal or multiportal wedge resections with VATS

Characteristic	Uniportal *N*=16	Multiportal *N*=55	*P*
Age (year)	59.44±21.00	55.67±20.36	0.5312
Sex (male/female)	13/3	34/21	0.2304
Median height (cm)	168.06±9.43	163.82±9.80	0.1289
Weight (kg)	66.67±15.04	59.25±12.83	0.0869
Brinkman Index	536.25±491.34	486.30±965.97	0.7814
Comorbidity			
Diabetes	2 (12.50)	6 (10.91)	1.0000
COPD	1 (6.25)	2 (3.64)	0.5410
IP	1 (6.25)	3 (5.45)	1.0000
Preoperative FEV1.0 (L)	2.54±0.76	2.43±0.61	0.6802
Disease			0.1311
Lung cancer	5 (31.25)	13 (23.64)	
Metastatic lung cancer	5 (31.25)	17 (30.91)	
Spontaneous pneumothorax	4 (25.00)	16 (29.09)	
Benign tumors	2 (12.50)	5 (9.09)	
Interstitial pneumonia	0 (0.00)	3 (5.45)	
Intralobar sequestration	0 (0.00)	1 (1.82)	

Data are presented as *n* (%); or as median value±standard error. COPD: Chronic obstructive pulmonary disease; FEV1.0: Forced expiratory volume in 1 second; IP: Interstitial pneumonia; VATS: video-assisted thoracoscopic surgery.

**Table4:** Intraoperative and postoperative background information of patients who underwent uniportal or multiportal wedge resections with VATS

Characteristic	Uniportal *N*=16	Multiportal *N*=55	*P*
Duration of surgery (min)	81.81±38.03	85.95±36.22	0.3782
Volume of hemorrhage (ml）	13.13±27.58	9.85±12.44	0.3415
Intraoperative complication	0 (0.00)	1 (1.82)	1.0000
Duration of hospitalization (day)	7.06±2.35	8.27±6.40	0.9275
Duration of in situ pleural cavity drain (day)	1.38±0.89	1.58±1.77	0.9250
Duration of hospitalization postoperatively (day)	5.25±2.26	4.56±2.33	0.3010
NRS on 1^st^ day after surgery	1.56±1.50	2.87±2.39	0.0426
Postoperative complication	0 (0.00)	2 (3.64)	1.0000
Pulmonary fistula	0	2	
Repeated surgery	0 (0.00)	0 (0.00)	1.0000
Duration of follow-up (day)	237.25±168.71	181.14±212.41	0.3360

Data are presented as *n* (%); or as median value±standard error. NRS: Numerical Rating Scale; VATS: video-assisted thoracoscopic surgery.

**Table5:** Pain evaluation of patients who underwent uniportal or multiportal lobectomies and segmentectomies with VATS

Variable	Uniportal *N*=19	Multiportal *N*=133	*P*
NRS	0.26±0.73	0.68±1.47	0.1973
Administration period (day)	26.68±15.28	50.14±61.07	0.0917
Amount of postoperative oral analgesics (tablet)	65.58±41.13	149.33±170.96	0.0041
Post thoracotomy pain syndrome	3 (15.79)	58 (43.61)	0.0240

Data are presented as *n* (%); or as median value±standard error. NRS: Numerical Rating Scale; VATS: video-assisted thoracoscopic surgery.

**Table6:** Pain evaluation of patients who underwent uniportal or multiportal wedge resections with VATS

Variable	Uniportal *N*=16	Multiportal *N*=55	*P*
NRS	0.00±0.00	0.40±1.05	0.0864
Administration period (day)	18.50±6.46	37.90±58.86	0.2043
Amount of postoperative oral analgesics (tablet)	51.69±15.17	109.44±146.41	0.0898
Post thoracotomy pain syndrome	0 (0.00)	13 (26.64)	0.0315

Data are presented as *n* (%); or as median value±standard error. NRS: Numerical Rating Scale; VATS: video-assisted thoracoscopic surgery.
